# Ki-67 in endometrial cancer: scoring optimization and prognostic relevance for window studies

**DOI:** 10.1038/modpathol.2016.203

**Published:** 2016-12-02

**Authors:** Sarah Kitson, Vanitha N Sivalingam, James Bolton, Rhona McVey, Mashid Nickkho-Amiry, Melanie E Powell, Alexandra Leary, Hans W Nijman, Remi A Nout, Tjalling Bosse, Andrew G Renehan, Henry C Kitchener, Richard J Edmondson, Emma J Crosbie

**Affiliations:** 1 Division of Molecular and Clinical Cancer Sciences, Faculty of Biology, Medicine and Health, University of Manchester, St Mary's Hospital, Manchester, UK; 2 Department of Obstetrics and Gynaecology, Central Manchester University Hospitals NHS Foundation Trust, Manchester Academic Health Science Centre, Manchester, UK; 3 Department of Histopathology, Central Manchester University Hospitals NHS Foundation Trust, Manchester Academic Health Science Centre, Manchester, UK; 4 Department of Clinical Oncology, Barts Health NHS Trust, London, UK; 5 INSERM U981 and Department of Medicine, Gynecology Unit, Gustave Roussy, Villejuif, France; 6 Department of Gynecology, University of Groningen, University Medical Center Groningen, Groningen, The Netherlands; 7 Department of Clinical Oncology, Leiden University Medical Center, Leiden, The Netherlands; 8 Department of Pathology, Leiden University Medical Center, Leiden, The Netherlands

## Abstract

Ki-67, a marker of cellular proliferation, is increasingly being used in pre-surgical window studies in endometrial cancer as a primary outcome measure. Unlike in breast cancer, however, there are no guidelines standardizing its measurement and its clinical relevance as a response biomarker is undetermined. It is, therefore, imperative that Ki-67 scoring protocols are optimized and its association with patient survival rigorously evaluated, in order to be able to clinically interpret the results of these studies. Using the International Ki-67 in Breast Cancer Working Group guidelines as a basis, whole slide, hot spot and invasive edge scoring protocols were evaluated using endometrial biopsies and hysterectomy specimens from 179 women. Whole sections and tissue microarrays, manual and semi-automated scoring using Definiens Developer software were additionally compared. Ki-67 scores were related to clinicopathological variables and cancer-specific survival in uni- and multivariate analysis. Against criteria of time efficiency, intra- and inter-observer variability and consistency, semi-automated hot spot scoring was the preferred method. Ki-67 scores positively correlated with grade, stage and depth of myometrial invasion (*P*-values all <0.03). By univariate analysis, higher Ki-67 scores were associated with a significant reduction in cancer-specific survival (*P*≤0.05); however, this effect was substantially attenuated in the multivariate model. In conclusion, hot spot scoring of whole sections using Definiens is an optimal method to quantify Ki-67 in endometrial cancer window study specimens. Measured this way, it is a clinically relevant marker, though further work is required to determine whether reductions in Ki-67 in neoadjuvant intervention studies translate into improved patient outcome.

Despite the rising incidence and corresponding increase in deaths from endometrial cancer, there is a noticeable lack of research into new prevention and treatment strategies.^1, 2^ There is a dearth of high quality clinical trial evidence to inform the management of women with advanced or recurrent endometrial cancer. Similarly, there is a lack of robust evidence to guide the clinical care of women who are unfit for surgery or who desire fertility-sparing treatment.

Clinical trials require large numbers of participants over long follow-up periods to demonstrate the superiority of one treatment over another on clinically important outcomes, such as overall and cancer-free survival. In cancer types that are amenable to diagnostic sampling, novel interventions can be screened for efficiently using the pre-surgical window study design, whereby tissue endpoints are compared before (at diagnosis) and after treatment (at definitive surgery) using biomarkers as surrogates for clinical endpoints. Ideally such biomarkers should have prognostic utility and be able to predict response to adjuvant treatment and longer term outcome.^3^ Their use allows the rapid screening of new interventions so that time, effort, and financial resources can be directed at treatments that hold the most promise.

Window studies in endometrial cancer are hampered by the lack of validated biomarkers meeting these criteria. In breast cancer, the nuclear protein Ki-67 is an established prognostic and predictive biomarker.^4, 5, 6^ Expressed only during the active G_1_, S, and G_2_ phases of the cell cycle, its expression is a marker of cellular proliferation and is readily detected by immunohistochemistry.^7^ The International Ki-67 in Breast Cancer Working Group set standards for the staining, scoring and analysis of Ki-67 in breast cancer to ensure the reproducibility, reliability and accuracy of studies using Ki-67 as their primary outcome measure.^8^ In brief, these included:

- Sole use of the MIB-1 antibody with heat-induced epitope retrieval
- Inclusion of positive and negative controls in all batches
- Scoring at least three high-power fields (× 40 magnification) across whole sections, incorporating the invasive edge of the tumor and hot spots
- Assessment of nuclear staining only (intensity of staining not relevant)
- Counting at least 500 (and preferably 1000) malignant cells
- Expressing the Ki-67 score as the percentage of positively stained cells among the total number of malignant cells assessed

Despite ambiguity in the literature about the value of Ki-67 as a biomarker in endometrial cancer, pre-surgical window studies using a change in Ki-67 as their primary endpoint have begun in earnest.^9, 10, 11, 12, 13^ Although Ki-67 expression has been shown to positively correlate with tumor grade,^12, 14, 15, 16^ there is a lack of consensus as to whether it has prognostic value.^14, 15, 16, 17, 18^ Heterogeneity of staining, scoring, and analysis protocols, including the use of study-specific cut-off values, have also hampered the validation of findings in other cohorts and, by extension, hindered the clinical interpretation of results from the aforementioned window studies. Furthermore, most previous studies were published over 10 years ago, using the now superseded FIGO 1988 staging criteria, limiting their applicability to modern clinical research.^17, 18^

The aims of this study were three-fold: to identify the most reliable, reproducible and time efficient method of Ki-67 scoring using the recommendations of the International Ki-67 in Breast Cancer Working Group as a guide;^8^ to determine the correlation between Ki-67 and known pathological prognostic variables; and to investigate whether higher Ki-67 expression is associated with a shorter cancer-specific survival and, therefore, has clinical value as a biomarker in endometrial cancer trials.

## Materials and methods

### Patient and Tissue Selection

The study was designed, analyzed, and reported in accordance with the REMARK guidelines for tumor marker prognostic studies.^19^ Tumor tissues from 179 patients undergoing hysterectomy for endometrial cancer were retrospectively selected. This included 128 consecutive patients who had donated tissue for research to the Manchester BRC Biobank from 2009 to 2014. Due to a preponderance of low grade and stage disease in this cohort, an additional 51 high-risk patients, for whom tissue and clinical follow-up data were available, were included from partner institutions of the TransPORTEC consortium (Leiden University Medical Center, The Netherlands; University Medical Center Groningen, The Netherlands; University College London, United Kingdom; and Gustave Roussy Paris, France) to ensure a representative population. This latter group included patients who had undergone primary surgery between 1991 and 2010. All grades, stages, and histological subtypes of endometrial cancer were included. All patients underwent surgery. Patients with intermediate or high-risk disease were given adjuvant treatment according to local protocols.

Tumor from hysterectomy specimens and, for a subset of tumors, corresponding endometrial biopsies taken immediately before the start of surgery, were formalin fixed and paraffin embedded, and stored at room temperature for up to 24 years. Four-*μ*m thick sections were cut from representative paraffin blocks using a cryostat and mounted onto a histological glass slide. Slides were either stained immediately or stored at +4 °C pending immunohistochemistry. Whole hematoxylin and eosin-stained slides were reviewed by experienced gynaecological histopathologists (JB, RM, and TB) to confirm FIGO (2009) stage, histological subtype, grade, depth of myometrial invasion and the presence or absence of lymphovascular space invasion. Tissue microarrays were created by the study histopathologists from hysterectomy specimens for a subset of the Manchester patients and the transPORTEC cohort using triplicate tumor cores. This allowed the effect of slide preparation technique to be determined by comparing Ki-67 scores from whole sections and tissue microarrays obtained from the same tumor.

### Immunohistochemistry

Immunohistochemistry was performed using the Leica Bond Max (Leica Biosystems, Wetzlar, Germany) with heat-induced epitope retrieval. This fully automated system is routinely used in many hospitals and ensures consistent staining across runs. Staining was performed using the optimized protocol recommended by the International Ki-67 in Breast Cancer Working Group.^8^ Antigen retrieval was undertaken at pH 9 for 20 min. A casein block of 30 min duration was carried out to reduce non-specific antibody binding. Slides were incubated at room temperature for 1 h with the MIB-1 antibody (monoclonal mouse, anti-human Ki-67 antibody; DAKO, Carpinteria, CA), at a dilution of 1:100. Primary antibody detection was undertaken using the Refine Detection Kit (Leica Biosystems), which contains a rabbit anti-mouse IgG secondary antibody and anti-rabbit poly-HRP IgG antibody and utilizes 3,3′-diaminobenzidine as a chromogen. Slides were counterstained with hematoxylin. Negative (isotype control) and positive (tonsil) controls were used for quality assurance.

### Ki-67 Scoring

**Figure 1:**
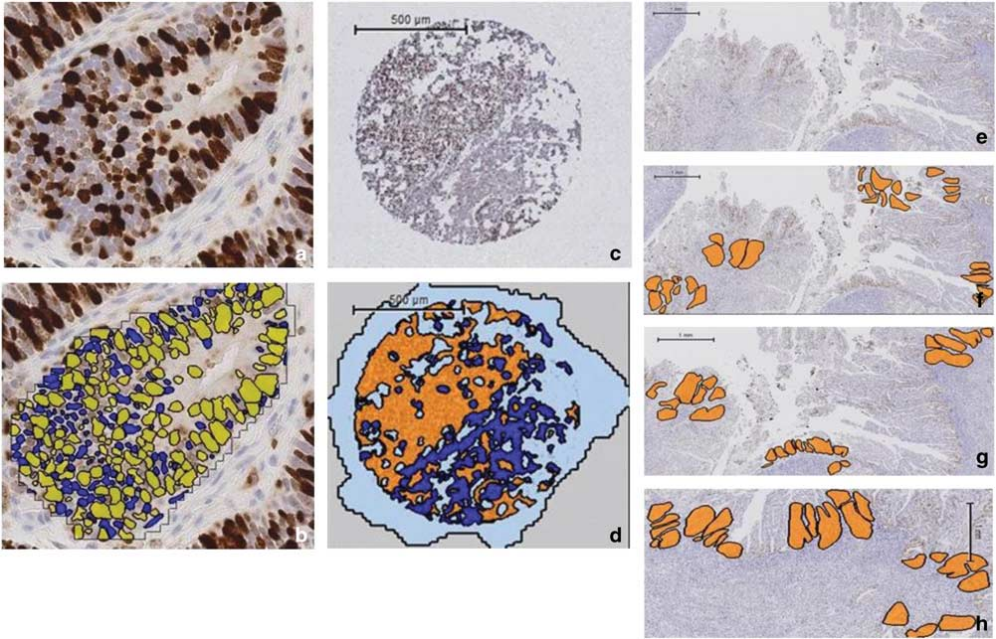
Ki-67 immunohistochemistry and scoring using Definiens Developer on whole sections and tissue microarrays. The accuracy of the solution to correctly identify individual positively and negatively stained nuclei was manually checked by comparing individual endometrial cancer glands with and without the solution applied. (**a**) Photomicrograph of endometrial cancer gland with Ki-67 immunohistochemistry applied (**b**) digital scoring output (× 20 magnification). Positively stained nuclei are yellow, negatively stained nuclei are blue. (**c**) Representative tissue microarray core following Ki-67 immunohistochemistry. (**d**) Same tissue microarray core following the application of Definiens Developer solution, with endometrial cancer glands shown in orange and the surrounding stroma in dark blue. Areas of the slide without the presence of tissue are colored pale blue. (**e**) Whole section of tumor following Ki-67 immunohistochemistry. Compared with the tissue microarray, a significantly greater tumor area is present on a whole section and is a better representation of the heterogeneity in proliferation seen across endometrial cancers. (**f**) The same section of tumor with the five areas selected at random using Definiens Developer software highlighted in orange to determine the whole slide score. (**g**) Three areas of greatest proliferation identified to provide a hot spot score. (**h**) Three areas along the endometrial/myometrial interface to quantify the invasive edge score.

Slides were digitized using the Leica SCN400 Slide Scanner (Leica Microsystems, Wetzlar, Germany). A semi-automated score was obtained by applying a computerized algorithm (Definiens Developer) to the malignant glands (Figure 1a and b). Manual selection of malignant glands guaranteed that scoring was limited to these areas and that stromal and inflammatory cells were excluded. In the case of carcinosarcomas, only malignant glands (the carcinoma component) were selected for scoring. Manual selection of malignant glands was repeated prior to each application of the algorithm. Malignant glands were visually compared prior to and following application of the Definiens Developer solution to ensure the correct classification of nuclei as positively and negatively stained and that debris and artifact were reliably excluded. All stained nuclei were counted as positive, irrespective of staining intensity. Different algorithms were tried and their accuracy checked for whole section and tissue microarray analyses, although similar rules and thresholds applied. For each algorithm, the accuracy of nuclei detection was confirmed using a subset of 12 randomly selected slides. For whole slides, the Ki-67 proliferation index, referred to hereafter as the Ki-67 score, was the percentage of positively stained nuclei scored according to three methods: whole slide, hot spot, and invasive edge scoring. For manual scoring, the percentage of positively stained nuclei within three high-powered fields (× 40 magnification) randomly selected across the tumor was calculated, ensuring at least 1000 nuclei were counted. Using the semi-automated system, all nuclei within three (hot spot and invasive edge) or five (whole slide) representative high-powered fields (× 20) were scored (at least 2000 nuclei in total). The areas to be scored were selected randomly across the section to take into account the heterogeneous proliferation seen in endometrial tumors (whole slide scoring), from areas of maximal Ki-67 staining (hot spot scoring) or from the endometrial/myometrial interface (invasive edge scoring) by two independent scorers (SK and VS), who were blinded to patient outcome (Figure 1c–h). For the tissue microarrays, all malignant glands of each tumor core were scored in their entirety.

Individual tumor cores and full sections were scored three times (twice by SK, once by VS) and the final Ki-67 score was calculated as the mean value of the three repeats. For the tissue microarrays, the final Ki-67 score for each tumor was the average of nine measurements; three cores from each tumor scored on three separate occasions. Discordant results of >10% (between SK and VS) were settled by consensus. The time to score individual slides was measured using a stopwatch.

### Follow-up Data Collection

Demographic, pathology, and follow-up data were obtained from electronic and hard copy patient records. In Manchester, patients were reviewed in specialist clinics every 4 months for the first two years and six monthly thereafter for a total of five years. The detection of recurrent disease was by way of symptom enquiry and clinical examination, with imaging as required. Cause of death was determined from primary care and mortuary records. For the transPORTEC patients, clinical follow-up data were provided by individual clinicians and stored in a secure database. All cases without events were censored at the last follow-up visit.

### Statistical Analysis

Tumor availability and consent for follow-up data collection limited the sample size to 179 patients; similar numbers to previous studies of Ki-67 in endometrial cancer.^14, 15, 18^ Importantly, this cohort included 26 endometrial cancer-related deaths and 41 recurrences, ensuring that the study was adequately powered to investigate the effect of Ki-67 on endometrial cancer recurrence and survival.^19^

**Figure 2:**
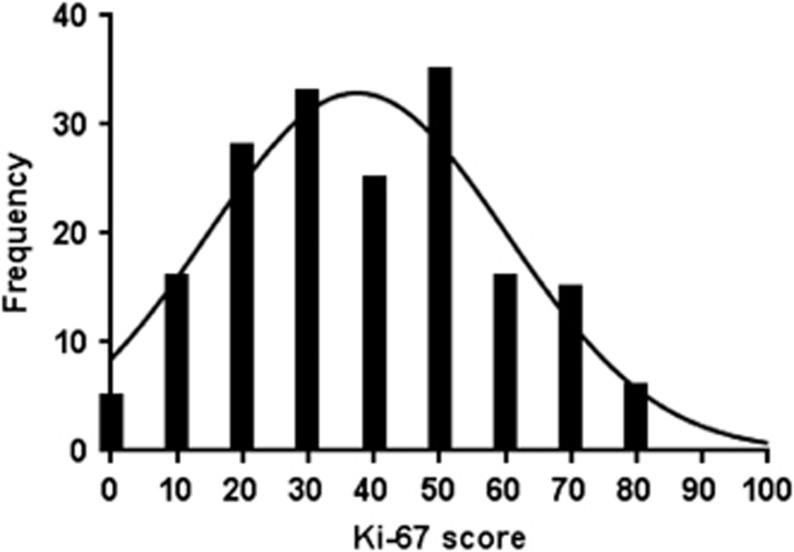
Frequency distribution of Ki-67 scores, as measured by the hot spot scoring method in 179 patients. The median Ki-67 score was 40%, with an interquartile range of 24–52%.

Ki-67 was measured as a continuous score using the hot spot method and data conformed to a negatively skewed distribution (Figure 2). Intra- and inter-observer variability was assessed by intra-class correlation coefficient. Bland–Altman plots were constructed to compare scores from endometrial biopsies and corresponding hysterectomy specimens and different slide preparation techniques, with 95% limits of agreement interpreted clinically. The association between Ki-67 and other pathological and clinical variables was tested using the Mann–Whitney *U*-test for non-parametric data and Spearman rank correlation for continuous and ordinal variables. Kaplan–Meier curves were constructed to estimate cancer-specific survival according to Ki-67 score and the log-rank test for trend used to compare curves. Cancer-specific survival was defined as the time between date of surgery and death from endometrial cancer. Recurrence-free survival was the interval between date of surgery and first documentation of recurrent disease. A Cox proportional hazard regression model was used in uni- and multivariate analyses of cancer-specific and recurrence-free survival, after confirming that the data complied with the proportional hazards assumption using log–log curves. These analyses examined Ki-67 as a continuous variable, using 10% increments to derive hazard ratios. The univariate analysis included previously documented important co-variates; age, body mass index (<30 kg/m^2^ *vs* ≥30 kg/m^2^), grade (1, 2, and 3), stage (1, 2, 3, and 4), histological type (endometrioid *vs* non-endometrioid), lymphovascular space invasion (presence *vs* absence), depth of myometrial invasion (<50% *vs* ≥50%), and adjuvant therapy use (yes *vs* no). The multivariate analysis utilized the significant prognostic variables identified in the univariate analysis. The model was developed using forward stepwise regression and confirmed using backward stepwise regression. Both methods produced identical results. A *P*-value of ≤0.05 was regarded as being of statistical significance. The statistical analysis was carried out using SPSS version 22 and GraphPad Instat.

## Results

### Optimization of Ki-67 Scoring

The semi-automated platform, combined with whole slide and hot spot scoring methods, demonstrated excellent intra- and inter-observer agreement, comparable to that seen with manual scoring (Table 1). The intra-class correlation coefficient values of 0.906–0.962 correspond to ‘almost perfect' agreement between repeated measurements by the same and different observers. Invasive edge scoring, in contrast, had lower reproducibility (intra-class correlation coefficient 0.750–0.868) and could only be performed on the 50% of available slides in which the endometrial/myometrial interface was sampled, limiting the value of this scoring method. Semi-automated scoring was considerably more time efficient than manual scoring, saving over 4 min per slide (2.2–3.1 min *vs* 7.7 min).

**Figure 3:**
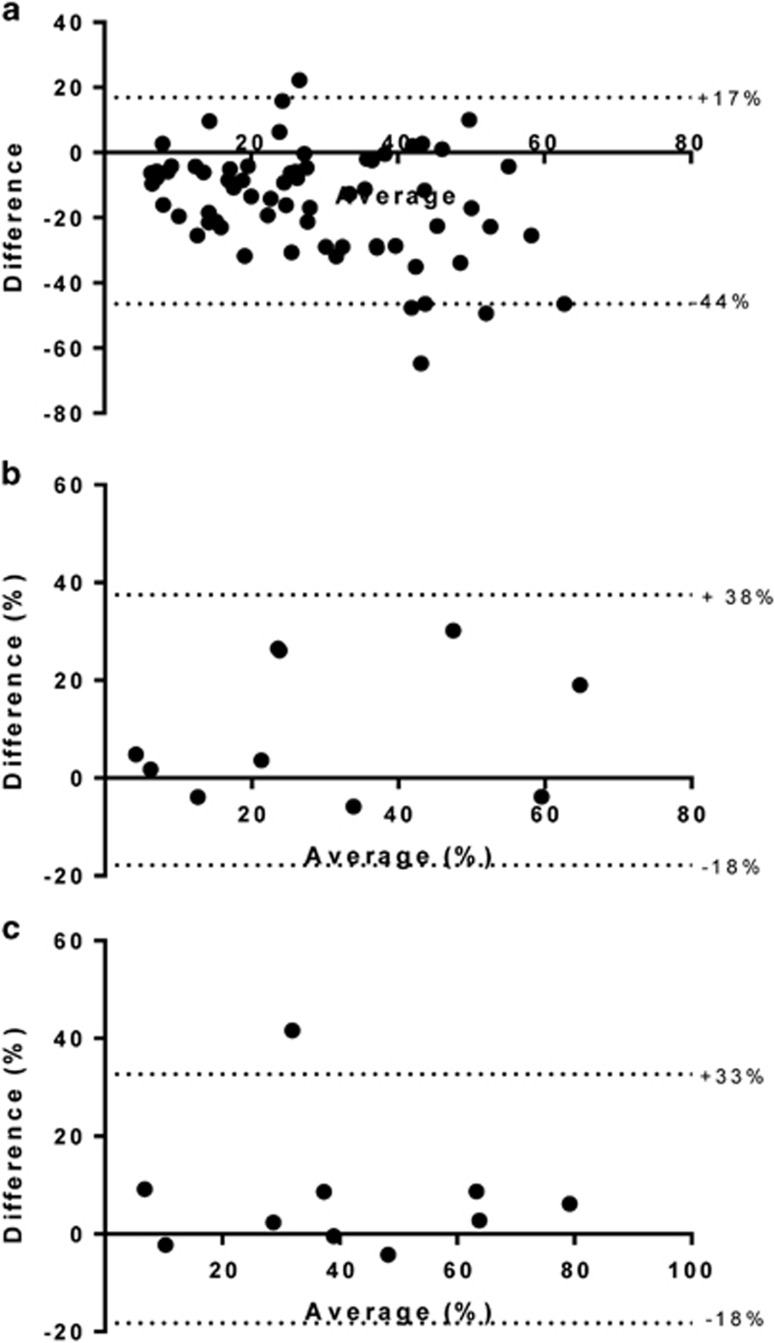
Comparison of different slide preparation and tumor-sampling techniques. Each point shows the difference between techniques plotted against the average of the two values. (**a**) Tissue microarray *vs* whole section from same tumor. Significant discrepancy was noted between tissue microarray and whole sections scores, with 95% limits of agreement lying at +17% and −44%. Endometrial cancer specimens were obtained from the same patient by blinded endometrial sampling performed immediately prior to surgery (pipelle) and after the uterus had been surgical removed (hysterectomy). (**b**) Whole slide scoring method. Significant variation between the two tumor-sampling techniques was noted using whole slide scoring (95% limits of agreement −18 to +38%). (**c**) Hot spot scoring method. In contrast, hot spot scoring appeared more consistent, with the exception of a single outlier.

Whole slides and tissue microarrays from the same tumor were available for a subset of the Manchester patients (*n*=17) and 50 of the 51 TransPORTEC patients. In general, there was poor agreement between whole slide and tissue microarray scores for individual patients (Figure 3a and Table 2), particularly when slides had been cut at the same time but stained several months apart. Delayed staining (of 3 months or more) resulted in much lower Ki-67 scores (data not shown). Within tissue microarrays, there was substantial variation in scores between individual cores from the same tumor and between observers (inter-observer intra-class correlation coefficient 0.701).

As the window study design necessitates analysis of tumor tissue prior to and following pre-surgical intervention, the consistency of Ki-67 scores across different tumor sampling techniques is important. Scores determined using the whole slide scoring method and Definiens software varied significantly between endometrial biopsies taken immediately prior to surgery and the corresponding hysterectomy specimen (Figure 3b, 95% limits of agreement −18 to +38%). Hot spot scoring (Figure 3c) was more consistent, with the exception of a single outlier, which, when removed, reduced the 95% limits of agreement to –7 to 13%. On the basis of these findings, hot spot scoring was deemed the optimal scoring method and was applied in survival analyses to determine the clinical relevance of Ki-67.

### Clinical Relevance of Ki-67

The cohort included 116 endometrioid and 63 non-endometrioid type (including serous, clear cell, carcinosarcoma, mixed, and undifferentiated) cancers, of which 108 were FIGO stage 1 (60%), 22 were stage 2 (12%), 42 were stage 3 (24%), and 6 were stage 4 (3%). The estimated median follow-up time, using the reverse Kaplan–Meier method, was 39.5 months, during which time 41 (23%) patients had local (22, 12%) and/or distant recurrences (35, 20%). There were 47 deaths (26%), of which 26 (15%) were from endometrial cancer. For grade 1/2 endometrioid, grade 3 endometrioid and non-endometrioid type cancers, 5-year cancer-specific survival rates were 93%, 88%, and 43% (*P*<0.0.001), respectively.

The median Ki-67 score in the overall cohort was 40%, with an interquartile range of 24–52%. The relationship between Ki-67 and patient clinicopathological characteristics was investigated (Table 3). As expected, Ki-67 score was closely associated with tumor grade (*P*≤0.001). In addition, it was also positively correlated with patient age, stage, depth of myometrial invasion, and adjuvant therapy use (*P*-values all ≤0.04). Scores were higher in those tumors with lymphovascular space invasion present and non-endometrioid histology, though these results did not reach statistical significance.

**Figure 4:**
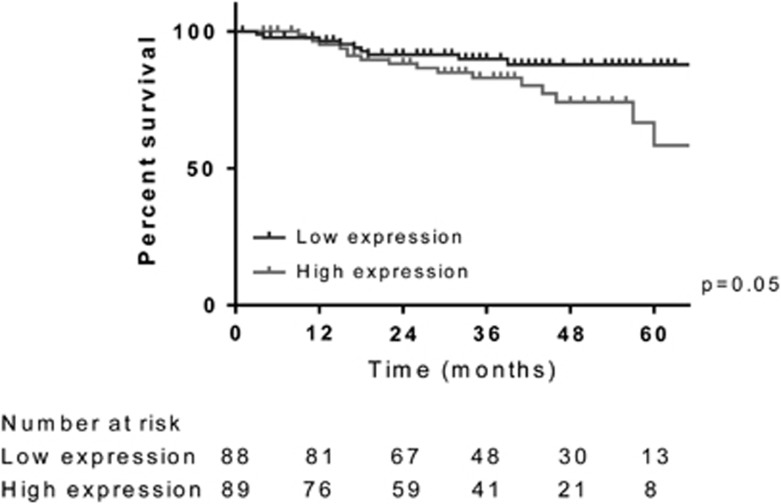
Cancer-specific survival stratified by Ki-67 expression. Ki-67 score was divided into two groups using the median score of 40% to denote low (Ki-67 score ≤40%) and high (Ki-67 score >40%) expression. It demonstrated a significant relationship with cancer-specific survival, with outcome worsening as the Ki-67 score increased. At 5 years, a Ki-67 score ≤40% was associated with cancer-specific survival rate of 88% compared with 58% for those with a Ki-67 score >40% (*P*=0.05).

Ki-67 scores were divided into two equal groups using the median score of 40% to denote low and high expression, to explore the relationship between Ki-67 and cancer-specific survival. The Kaplan–Meier curves suggested that greater tumor proliferation was associated with a significant reduction in survival; 5-year cancer-specific survival rates were 58% for those tumors with high Ki-67 expression, compared with 88% for those with tumors with low Ki-67 expression (Figure 4, *P*=0.05).

In a univariate analysis, Ki-67 score, as a continuous variable, as well as age, grade, stage, and histological type of endometrial cancer, presence or absence of lymphovascular space invasion and depth of myometrial invasion, was a prognostic indicator of cancer-specific survival (Table 4). A 10% increase in Ki-67 was associated with a 31% (95% CI 7–60%) worsening of cancer-specific survival. After adjustment for important clinicopathological variables and Ki-67 score, only age, stage and histological type of endometrial cancer remained independent prognostic variables for cancer-specific survival (Table 3). Ki-67 failed to reach statistical significance in the multivariate analysis.

Analyses were repeated using recurrence-free survival as the outcome of interest and produced similar results.

## Discussion

This is the first study to compare semi-automated scoring using Definiens Developer software with manual Ki-67 scoring in endometrial cancer. Although unable to differentiate between malignant glands and stromal tissue, when the areas to be scored were manually selected by observers blinded to outcome, the accuracy and reproducibility of scoring by Definiens was extremely high. Automated scoring was superior to manual scoring in terms of speed and it was reliable across time and between scorers.

Compared with whole slide and invasive edge scoring, hot spot scoring was the most reproducible scoring method for Ki-67, with excellent intra- and inter-observer agreement, and the most consistent across different endometrial tumor-sampling techniques. This is of particular importance for window studies using Ki-67 as a primary outcome measure, where an endometrial biopsy taken prior to intervention is frequently compared with the hysterectomy specimen at the end of treatment to determine response.

The scoring of whole slides was found to be superior to that of tissue microarrays in terms of both reproducibility and consistency. There are no published comparisons of Ki-67 assessment by tissue microarray and whole slide scoring in the breast cancer literature for guidance, but the International Ki-67 in Breast Cancer Working Group do note anecdotal evidence for lower scoring on tissue microarrays and advise avoiding their use when establishing quantitative relationships with clinical outcomes.^8^ A study in ovarian cancer similarly showed that Ki-67 staining of tissue microarray cores may not be representative of the results obtained from whole section immunohistochemistry.^20^ Appreciation of the heterogeneity of staining seen within whole sections of endometrial tumors is lost when only a small area is sampled in a core, reflected in the poor correlation of scores. This becomes even more evident if there is a time interval between slides being cut and stained; a delay in staining of more than 6 weeks resulted in lower Ki-67 scores. This has previously been described for sections stored under varying conditions; even at 4 °C the resulting hydrolysis negatively impacts on antigenicity.^21^ The authors, therefore, recommend undertaking staining on freshly cut sections to avoid this problem and limiting assessment to whole sections only. If freshly cut sections are not available, it is important that all slides are cut at the same time and later stained together for accurate comparison within a study, with the caveat that this limits comparability between studies.

Using the optimized methodology of semi-automated hot spot scoring, Ki-67 score was strongly associated with known pathological prognostic variables, including grade, stage, and depth of myometrial invasion. Although not independent of other prognostic factors, high Ki-67 was associated with poor cancer outcomes. These data are consistent with those of Salvesen *et al*,^22^ Stefansson *et al*,^14^ Geisler *et al*,^18^ and Liu *et al*,^15^ who described Ki-67 as a prognostic biomarker in endometrial cancer, although significance was generally lost after adjusting for important pathological variables like grade of disease and histological subtype. These studies were considerably larger than those of Fanning *et al*^17^ and Huvila *et al*,^16^ who published conflicting results; the latter studies had fewer disease events, shorter follow-up periods and were fundamentally underpowered to detect a significant effect of Ki-67 on cancer-specific outcomes.

Detailed clinical follow-up and expert pathology review are strengths of this study. The included population was sufficiently large to ensure that the study was adequately powered; 10–25 events are required per prognostic variable under investigation.^19^ Meticulous documentation of date of recurrence and cause of death allowed cancer-specific and recurrence-free survival to be calculated, arguably more clinically relevant endpoints than the overall survival used in other studies.^16, 18^

The median Ki-67 score was similar to that of other studies (40% *vs* 33–40%),^14, 15, 18^ who used median Ki-67 to dichotomize tumors into low and high Ki-67 expression. This approach is crude and prevents extrapolation across study populations. Ours is the first study to consider Ki-67 as a continuous variable, equating a 10% increase in Ki-67 expression with a cancer-specific survival hazard ratio of 1.31. This information is important for clinical trials using Ki-67 as a primary endpoint as it provides some degree of clinical context in which to interpret the results. The magnitude of effect seen in this study is similar to that shown in breast cancer studies, where Ki-67 expression is routinely log transformed to normalize the data. In breast cancer, the hazard ratio per 2.7-fold increase in Ki-67 expression was 1.95 for recurrence-free survival.^23^ Applying the same methodology to our findings for ease of comparison, the hazard ratio for recurrence-free survival in endometrial cancer was 1.94 (95% CI 1.10–3.43).

These findings are unsurprising, given that cancer is a disorder of unregulated cell proliferation.^24^ When measured by different methodologies, including S-phase fraction by flow cytometry, immunohistochemical staining of proliferative cell nuclear antigen, Ki-67 or manual counting of mitotic figures, cell proliferation increases across the spectrum of endometrial cancer development, from normal endometrium through to hyperplasia and cancer, with the highest rates seen in grade 3, serous, and clear cell cancers.^25, 26^ It is also closely associated with tumor grade and stage, known important prognostic variables in endometrial cancer.^15, 27^ It is logical to hypothesize, therefore, that those cancers with the greatest cell proliferation will have the poorest clinical outcome and that the fastest dividing areas of the tumors (the hot spots) will be closely associated with disease metastasis and recurrence.

A limitation of this study was that too few tumors were available to adequately power the assessment of Ki-67 in a multivariate analysis, controlling for all known prognostic clinicopathological variables. The aim of this study, however, was not to ascertain whether Ki-67 could replace pathological prognostic variables, but rather to determine its value as a primary tissue endpoint for use in clinical trials, where the window is of treatment is too short to observe changes in grade and stage of disease. In breast cancer, a drop in Ki-67 following short-term treatment with neoadjuvant chemotherapy predicts long-term response to that drug in the adjuvant setting.^6^ Our data suggest that Ki-67 could be used to stratify patients for entry into endometrial cancer adjuvant drug trials, excluding those whose prognosis is so good that they are unlikely to derive benefit from further therapeutic intervention beyond surgery. This is tentative speculation that requires formal testing. Ideally, this should include testing the same novel therapy before and after surgery and assess changes in pre-surgical Ki-67 score alongside longer-term cancer-specific and recurrence-free survival as outcome measures. Response to treatment could then be stratified according to baseline Ki-67 score. Such data are clearly required for drugs like metformin, which is increasingly being investigated in endometrial cancer window studies, if there is to be sufficient evidence of clinical efficacy for them to be used in routine practice.

In conclusion, these data provide evidence that semi-automated scoring of Ki-67 using Definiens Developer software is reliable, reproducible, and more time efficient than manual scoring and that hot spot methodology should be employed in future clinical trials as it is the most consistent across endometrial biopsies and hysterectomy specimens. When measured using standardized protocols of immunohistochemical staining, Ki-67 is associated with endometrial cancer survival and is, therefore, a clinically relevant endpoint, though further work is required to determine whether it fulfills all of the criteria to be used as a biomarker of treatment response.

## Figures and Tables

**Table 1:** Comparison of manual and semi-automated scoring of Ki-67 expression

*Criteria*	*Manual scoring* n=*33*	*Semi-automated scoring* n=*179*		
		*Whole slide scoring*	*Hot spot scoring*	*Invasive edge scoring*
Intra-observer intra-class correlation coefficient	0.91	0.96	0.96	0.75
Inter-observer intra-class correlation coefficient	0.92	0.92	0.91	0.87
Percentage of slides possible to score in cohort	100	100	100	50
Time per slide (min)	7.7	3.1 (+2 min to run solution)	2.3 (+2 min to run solution)	2.2 (+2 min to run solution)

Invasive edge scoring had lower intra- and inter-observer reproducibility than manual, whole slide or hot spot scoring and could only be performed on the 50% of available slides in which the endometrial–myometrial interface was sampled. Manual scoring had ‘almost perfect' intra- and inter-observer reproducibility but took considerably longer to perform than whole slide and hot spot scoring using the Definiens Developer algorithm.

**Table 2:** Comparison of tissue microarray and whole slide scores for matched tumors

	*Tissue microarray*	*Whole slide*
Number of matched tissue microarray and whole slide sections	*n*=67^a^	
Median Ki-67 score (interquartile range)	19% (10–31%)	32% (21–47%)
Number of sections in which the tissue microarray and whole section scores were within 10% of each other	*n*=28 (42%)	

a Tissue microarrays were constructed from the same blocks as those used to cut whole slides and therefore have overlapping areas of tumor.

**Table 3:** Relation between patient characteristics and Ki-67 score

*Characteristic*	*Number of patients (%)*	*Median Ki-67 score % (interquartile range)*	P*-value*
All	179	40 (24–52)	
			
*Age at diagnosis*			0.022*
Median (years)	68		
Interquartile range (years)	60–74		
			
*Body mass index (kg/m*^*2*^)			0.663
<25	22 (12)	44 (26–58)	
25–29.9	35 (20)	28 (17–47)	
≥30	64 (36)	41 (26–51)	
Missing data	58 (32)	43 (28–55)	
			
*Grade*			
1	39 (22)	23 (10–39)	<0.0001^****^
2	36 (20)	41 (26–50)	
3	104 (58)	44 (30–55)	
			
*Stage*			0.014^**^
1	108 (60)	36 (22–50)	
2	22 (12)	37 (24–55)	
3	42 (24)	44 (29–56)	
4	6 (3)	54 (45–67)	
Missing data	1 (1)	17 (17–17)	
			
*Histological type*			0.246
Endometrioid	116 (65)	37 (23–51)	
Non-endometrioid^a^	63 (35)	44 (28–54)	
			
*Lymphovascular space invasion*			0.138
Absent	93 (52)	37 (22–50)	
Present	70 (39)	42 (25–55)	
Missing data	16 (9)	45 (31–53)	
			
*Depth of myometrial invasion*			0.030*
<50%	83 (46)	32 (20–50)	
≥50%	92 (51)	43 (28–53)	
Missing data	4 (2)	42 (33–51)	
*Any adjuvant therapy*			0.031*
No	61 (34)	32 (22–49)	
Yes^b^	102 (57)	44 (27–55)	
Missing data	16 (9)	35 (26–45)	

Ki-67 expression positively correlated with age at the time of surgery, grade, and stage of endometrial cancer. Higher scores were seen in tumors with >50% myoinvasion compared with more superficially invasive cancers and women receiving adjuvant therapy. There was no association between Ki-67 and body mass index, histological type of endometrial cancer, and lymphovascular space invasion (*P*>0.05).

a Grade 3 endometrioid tumors are classified within the endometrioid subtype. Non-endometrioid tumors include serous, clear cell, carcinosarcomas, mixed, and undifferentiated cancers.

b Adjuvant treatment included external beam radiotherapy (44, 25%), vaginal brachytherapy (21, 12%) or both (25, 14%), and/or chemotherapy (39, 22%), which was single-agent carboplatin (2, 1%) or carboplatin/paclitaxel-based (29, 16%). Data on chemotherapy regime absent in 8 (5%) of cases. *p≤0.05, **p≤0.01, ***p≤0.001, ****p≤0.0001.

**Table 4:** Univariate and multivariate analysis of associations between Ki-67 score and standard variables and cancer-specific survival in 179 women with endometrial cancer

*Variable*	*Univariate analysis*			*Multivariate analysis*		
	*HR*	*95% CI*	P*-value*	*HR*	*95% CI*	P*-value*
Age (1 year)	1.10	1.05–1.15	<0.0001^****^	1.06	1.001–1.13	0.028*

*Body mass index*						
<30 kg/m^2^	1.00	—	0.52	—	—	—
≥30 kg/m^2^	1.51	0.43–5.25				

*Grade*						
1	1.00	—	0.014^**^	—	—	—
2	0.584	0.05–6.44				
3	4.975	1.17–21.12				

*Stage*						
1	1.00	—	<0.0001^****^	1.00	—	0.004^**^
2	0.96	0.12–7.99		3.27	0.34–31.78	
3	7.8	3.03–20.11		6.44	2.07–20.06	
4	23.36	6.49–84.05		16.09	2.83–91.36	

*Histological type*						
Endometrioid	1.00	—	<0.0001^****^	1.00	—	0.002^**^
Non-endometrioid	8.16	3.27–20.39		5.72	1.93–16.95	

*Lymphovascular space invasion*						
Absent	1.00	—				
Present	4.54	2.21–9.33	<0.0001^****^	—	—	—
*Depth of myometrial invasion*						
<50%	1.00	—		—	—	—
≥50%	2.46	1.03–5.90	0.043*			

*Any adjuvant therapy*						
No	1.00	—	0.202	—	—	—
Yes	2.05	0.68–6.19				
Ki-67 score (10% increase)	1.31	1.07–1.60	0.010^**^	1.14	0.91–1.41	0.257

Abbreviations: CI, confidence interval; HR, hazard ratio.

Ki-67 was no longer statistically significantly associated with cancer-specific survival when included in the multivariate analysis.

Variables found to be statistically significantly associated with cancer-specific survival in the univariate analysis (*P*≤0.05) were included in the multivariate analysis; ‘—' represents not statistically significant in the multivariate analysis. *p≤0.05, **p≤0.01, ***p≤0.001, ****p≤0.0001.

## References

[bib1] Cancer Research UKUterine (womb) Cancer Incidence Statistics 2014, Available at http://www.cancerresearchuk.org/health-professional/cancer-statistics/statistics-by-cancer-type/uterine-cancer/incidence (accessed 17 October 2014).

[bib2] Cancer Research UKUterine Cancer Mortality Statistics 2014, Available at http://www.cancerresearchuk.org/health-professional/cancer-statistics/statistics-by-cancer-type/uterine-cancer/mortality (accessed 17 October 2014).

[bib3] Cancer Research UKCRUK Prognostic/Predictive Biomarker (BM) Roadmap, Available at http://www.cancerresearchuk.org/prod_consump/groups/cr_common/@fre/@fun/documents/generalcontent/cr_027486.pdf (accessed 4 December 2015).

[bib4] Ellis MJ, Tao Y, Luo J et al, Outcome prediction for estrogen receptor-positive breast cancer based on postneoadjuvant endocrine therapy tumor characteristics. J Natl Cancer Inst 2008; 100: 1380–1388. 18812550 10.1093/jnci/djn309 PMC2556704

[bib5] de Azambuja E, Cardoso F, de Castro G Jr et al, Ki-67 as prognostic marker in early breast cancer: a meta-analysis of published studies involving 12,155 patients. Br J Cancer 2007; 96: 1504–1513. 17453008 10.1038/sj.bjc.6603756 PMC2359936

[bib6] Dowsett M, Smith IE, Ebbs SR et al, Short-term changes in Ki-67 during neoadjuvant treatment of primary breast cancer with anastrozole or tamoxifen alone or combined correlate with recurrence-free survival. Clin Cancer Res 2005; 11: 951s–958s. 15701892

[bib7] Scholzen T, Gerdes J. The Ki-67 protein: from the known and the unknown. J Cell Physiol 2000; 182: 311–322. 10653597 10.1002/(SICI)1097-4652(200003)182:3<311::AID-JCP1>3.0.CO;2-9

[bib8] Dowsett M, Nielsen TO, A'Hern R et al, Assessment of Ki67 in breast cancer: recommendations from the international Ki67 in Breast Cancer working group. J Natl Cancer Inst 2011; 103: 1656–1664. 21960707 10.1093/jnci/djr393 PMC3216967

[bib9] Mitsuhashi A, Kiyokawa T, Sato Y et al, Effects of metformin on endometrial cancer cell growth *in vivo*: a preoperative prospective trial. Cancer 2014; 120: 2986–2995. 24917306 10.1002/cncr.28853

[bib10] Laskov I, Drudi L, Beauchamp MC et al, Anti-diabetic doses of metformin decrease proliferation markers in tumors of patients with endometrial cancer. Gynecol Oncol 2014; 134: 607–614. 24972190 10.1016/j.ygyno.2014.06.014

[bib11] Schuler KM, Rambally BS, DiFurio MJ et al, Antiproliferative and metabolic effects of metformin in a preoperative window clinical trial for endometrial cancer. Cancer Med 2015; 4: 161–173. 25417601 10.1002/cam4.353 PMC4329001

[bib12] Sivalingam VN, Kitson S, McVey R et al, Measuring the biological effect of presurgical metformin treatment in endometrial cancer. Br J Cancer 2016; 114: 281–289. 26794276 10.1038/bjc.2015.453 PMC4742583

[bib13] Thangavelu A, Hewitt MJ, Quinton ND et al, Neoadjuvant treatment of endometrial cancer using anastrozole: a randomised pilot study. Gynecol Oncol 2013; 131: 613–618. 24076063 10.1016/j.ygyno.2013.09.023

[bib14] Stefansson IM, Salvesen HB, Immervoll H et al, Prognostic impact of histological grade and vascular invasion compared with tumour cell proliferation in endometrial carcinoma of endometrioid type. Histopathology 2004; 44: 472–479. 15139995 10.1111/j.1365-2559.2004.01882.x

[bib15] Liu T, Gao H, Yang M et al, Correlation of TNFAIP8 overexpression with the proliferation, metastasis, and disease-free survival in endometrial cancer. Tumour Biol 2014; 35: 5805–5814. 24590269 10.1007/s13277-014-1770-y

[bib16] Huvila J, Talve L, Carpen O et al, Progesterone receptor negativity is an independent risk factor for relapse in patients with early stage endometrioid endometrial adenocarcinoma. Gynecol Oncol 2013; 130: 463–469. 23777659 10.1016/j.ygyno.2013.06.015

[bib17] Fanning J, Brown S, Phibbs G et al, Immunohistochemical evaluation is not prognostic for recurrence in fully staged high-risk endometrial cancer. Int J Gynecol Cancer 2002; 12: 286–289. 12060450 10.1046/j.1525-1438.2002.t01-1-01103.x

[bib18] Geisler JP, Geisler HE, Miller GA et al, MIB-1 in endometrial carcinoma: prognostic significance with 5-year follow-up. Gynecol Oncol 1999; 75: 432–436. 10600302 10.1006/gyno.1999.5615

[bib19] Altman DG, McShane LM, Sauerbrei W et al, Reporting recommendations for tumor marker prognostic studies (REMARK): explanation and elaboration. PLoS Med 2012; 9: e1001216. 22675273 10.1371/journal.pmed.1001216 PMC3362085

[bib20] Khouja MH, Baekelandt M, Sarab A et al, Limitations of tissue microarrays compared with whole sections in survival analysis. Oncol Lett 2010; 5: 827–831. 10.3892/ol_00000145 PMC3436208 22966388

[bib21] Economou M, Schoni L, Hammer C et al, Proper paraffin slide storage is crucial for translational research projects involving immunohistochemistry stains. Clin Transl Med 2014; 3: 4. 24636624 10.1186/2001-1326-3-4 PMC3995437

[bib22] Salvesen HB, Iversen OE, Akslen LA. Prognostic significance of angiogenesis and Ki-67, p53, and p21 expression: a population-based endometrial carcinoma study. J Clin Oncol 1999; 17: 1382–1390. 10334522 10.1200/JCO.1999.17.5.1382

[bib23] Dowsett M, Smith IE, Ebbs SR et al, Prognostic value of Ki67 expression after short-term presurgical endocrine therapy for primary breast cancer. J Natl Cancer Inst 2007; 99: 167–170. 17228000 10.1093/jnci/djk020

[bib24] Hanahan D, Weinberg RA. The hallmarks of cancer. Cell 2000; 100: 57–70. 10647931 10.1016/s0092-8674(00)81683-9

[bib25] Ito K, Sasano H, Watanabe K et al, Immunohistochemical study of PCNA (proliferating cell nuclear antigen) in normal and abnormal endometrium. Int J Gynecol Cancer 1993; 3: 122–127. 11578332 10.1046/j.1525-1438.1993.03020122.x

[bib26] Morsi HM, Leers MP, Jager W et al, The patterns of expression of an apoptosis-related CK18 neoepitope, the bcl-2 proto-oncogene, and the Ki67 proliferation marker in normal, hyperplastic, and malignant endometrium. Int J Gynecol Pathol 2000; 19: 118–126. 10782407 10.1097/00004347-200004000-00004

[bib27] Prat J. Prognostic parameters of endometrial carcinoma. Hum Pathol 2004; 35: 649–662. 15188130 10.1016/j.humpath.2004.02.007

